# The Regulation of Sulfur Metabolism in *Mycobacterium tuberculosis*

**DOI:** 10.1371/journal.ppat.1002036

**Published:** 2011-07-21

**Authors:** Stavroula K. Hatzios, Carolyn R. Bertozzi

**Affiliations:** 1 Department of Chemistry, University of California, Berkeley, Berkeley, California, United States of America; 2 Department of Molecular and Cell Biology, University of California, Berkeley, Berkeley, California, United States of America; 3 Howard Hughes Medical Institute, University of California, Berkeley, Berkeley, California, United States of America; University of California San Diego, United States of America

## Abstract

*Mycobacterium tuberculosis* (*Mtb*) has evolved into a highly successful human pathogen. It deftly subverts the bactericidal mechanisms of alveolar macrophages, ultimately inducing granuloma formation and establishing long-term residence in the host. These hallmarks of *Mtb* infection are facilitated by the metabolic adaptation of the pathogen to its surrounding environment and the biosynthesis of molecules that mediate its interactions with host immune cells. The sulfate assimilation pathway of *Mtb* produces a number of sulfur-containing metabolites with important contributions to pathogenesis and survival. This pathway is regulated by diverse environmental cues and regulatory proteins that mediate sulfur transactions in the cell. Here, we discuss the transcriptional and biochemical mechanisms of sulfur metabolism regulation in *Mtb* and potential small molecule regulators of the sulfate assimilation pathway that are collectively poised to aid this intracellular pathogen in its expert manipulation of the host. From this global analysis, we have identified a subset of sulfur-metabolizing enzymes that are sensitive to multiple regulatory cues and may be strong candidates for therapeutic intervention.

## Introduction

*Mycobacterium tuberculosis (Mtb)*, the bacterium that causes tuberculosis in humans, infects roughly 2 billion people worldwide [1]. However, less than 1% of those infected with *Mtb* show signs of active disease [2]. The majority of infected individuals have a latent infection characterized by dormant, nonreplicating bacteria that persist within a mass of immune cells in the lung [3]. These cells form a protective barrier between the bacteria and surrounding tissue known as the granuloma [4], [5]. When host immunity is compromised, the granuloma deteriorates, liberating the confined bacteria and reactivating the disease.

In order to mount a latent infection, *Mtb* must withstand phagocytosis by alveolar macrophages, the host's primary line of defense against airborne pathogens [5]. By evading typical bactericidal processes, *Mtb* is able to replicate and eventually induce granuloma formation [3]. The mechanisms by which *Mtb* persists in the hostile phagosomal environment and orchestrates the transition to latency are ill defined. Elucidating the molecular machinery and metabolic pathways that facilitate these pivotal events is crucial to identifying new therapeutic targets for this global human pathogen.

**Figure 1:**
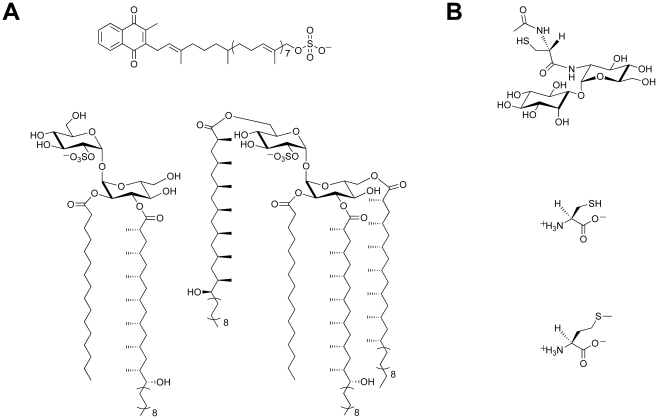
Sulfur-containing metabolites from *Mtb*. (A) Sulfated compounds, clockwise from top: S881, Sulfolipid-1, SL_1278_. (B) Reduced sulfur compounds, from top to bottom: MSH, cysteine, methionine.

There is a growing body of evidence that supports a role for sulfur-containing metabolites in *Mtb* pathogenesis [6], [7]. Sulfated molecules from *Mtb* have been intimately linked to bacterial virulence (Figure 1A) [8]–[10]. For example, the prominent cell wall-associated glycolipid Sulfolipid-1 is only produced by pathogenic species of mycobacteria [8], [11], and its biosynthetic precursor SL_1278_, named for its observed mass, has been shown to elicit cytokine production in human tuberculosis patients [10]. On the contrary, biosynthesis of the sulfated menaquinone S881 suppresses bacterial virulence [9]. Reduced sulfur compounds (Figure 1B), such as cysteine and methionine, also contribute to *Mtb* pathogenesis [6]. Disabling their biosynthesis dramatically attenuates bacterial virulence and persistence during the chronic phase of infection in mice [12]. Mycothiol (MSH), the primary thiol-containing small molecule of mycobacteria [13], is instrumental in the detoxification of numerous bactericidal agents and confers protection against oxidative stress [14]–[16]. Biosynthesis of these important sulfur-containing metabolites relies on the sulfate assimilation pathway [17].

**Figure 2:**
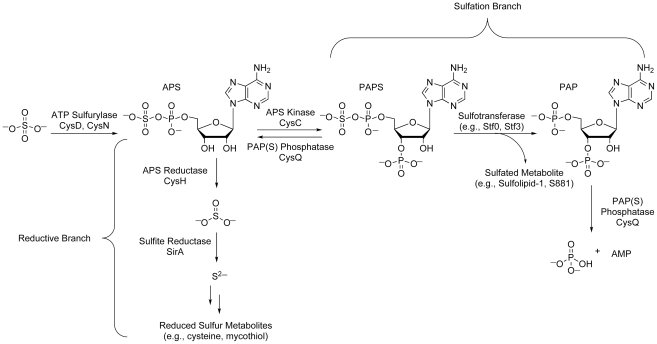
The sulfate assimilation pathway of *Mtb*. Inorganic sulfate from the host is converted to APS by ATP sulfurylase. APS is either sequentially reduced by APS reductase and sulfite reductase for the biosynthesis of essential reduced sulfur metabolites (reductive branch), or phosphorylated by APS kinase to generate PAPS, a substrate for sulfotransferases (sulfation branch). PAP, a byproduct of sulfotransferase reactions, is degraded by the PAP(S) phosphatase CysQ, which also converts PAPS to APS.

The sulfate assimilation pathway (Figure 2) is composed of a group of enzymes that catalyze the uptake and metabolism of inorganic sulfate from the host [7]. This pathway commences with the active import of sulfate, which is subsequently adenylated by ATP sulfurylase, an enzyme containing both GTPase and sulfurylase domains [18]. The resulting product, adenosine 5′-phosposulfate (APS), can be reduced to sulfite by APS reductase for the biosynthesis of reduced sulfur species via the reductive branch of the pathway [19]. Alternatively, because APS constitutes a metabolic branchpoint, it can be phosphorylated at the 3′-position by APS kinase to generate 3′-phosphoadenosine 5′-phosphosulfate (PAPS), the universal sulfate donor in the cell [17]. PAPS is a substrate for sulfotransferases, enzymes that catalyze the transfer of its sulfate group onto bacterial metabolites [11]. Collectively, these reactions constitute the sulfation branch of the sulfate assimilation pathway.

The regulation of *Mtb* sulfur metabolism relies on the transcriptional response of sulfate assimilation enzymes to diverse environmental cues and regulatory proteins that influence flux through the sulfate assimilation pathway. Further, small molecule regulation of sulfur metabolism in other bacteria suggests similar mechanisms may modulate related transcriptional circuits in *Mtb*. Here, we review each of these regulatory elements in an effort to guide our understanding of how the sulfate assimilation pathway facilitates bacterial adaptation to the host.

## Transcriptional Regulation of Sulfur Metabolism Genes

Transcriptional profiling has demonstrated that *Mtb* sulfur metabolism genes are dynamically regulated by diverse environmental cues (Table 1) [20]–[29]. This finding is perhaps not surprising, given the demonstrated importance of the sulfate assimilation pathway to bacterial survival [6] and the hostile conditions encountered by bacteria in the phagosome [30]. For example, upon phagocytosis, bacteria are exposed to oxidative stress and deprived of essential nutrients [30], [31]. Microarray analysis has shown that these conditions elicit the upregulation of key genes from the sulfate assimilation pathway (Table 1) [21], [22], [26], [27]. Genes encoding the primary sulfate transport complex of *Mtb* are induced following treatment with hydrogen peroxide (*cysT*) and nutrient starvation (*cysA1*, *cysT*, *cysW*, *subI*), conditions that also induce the ATP sulfurylase genes *cysD* and *cysN*, as well as the *cysC* gene that encodes APS kinase (in *Mtb*, *cysC* is fused to *cysN*, yielding a bifunctional *cysNC* gene [18]). Given that these genes coordinate the first few steps of the sulfate assimilation pathway, their induction is most likely accompanied by an increase in the biosynthesis of sulfur-containing metabolites that protect the pathogen during the course of infection, though this remains to be demonstrated. Notably, *cysD* and *cysNC* are also induced during macrophage infection, underscoring the importance of sulfur metabolism to intracellular survival [21], [25]. Indeed, reduced sulfur compounds have been shown to play a critical role in facilitating bacterial persistence *in vivo* [12]. Genes contributing to the biosynthesis of these metabolites, such as *cysH* and *sirA*, which direct the reduction of APS to sulfide [19], [32], and *cysM*, which is involved in cysteine biosynthesis [33], [34], are also among those induced by hydrogen peroxide and nutrient starvation. Collectively, these genes are likely to facilitate bacterial adaptation to the phagosomal environment.

**Table 1:** Sulfur metabolism genes from *M. tuberculosis* induced by various conditions of environmental stress.

Stress Condition	Genes Induced	Reference
5-Chloropyrazinamide	*cysN*	[20]
Hydrogen peroxide	*cysD*, *cysNC* a, *cysK2*, *cysM*, *cysT*	[21], [22]
Hypoxia	*cysD*, *cysK2*, *cysN*, *cysM*	[23], [24]
Macrophage infection	*cysD*, *cysN*	[21], [25]
Menadione	*cysA1*, *cysT*, *cysW*, *subI*	[20]
Nutrient starvation	*cysA1*, *cysD*, *cysH*, *cysN*, *cysQ*, *cysT*, *cysW*, *sirA*, *subI*	[26], [27]
SDS	*cysD*, *cysN*	[28]
Vancomycin	*cysD*, *cysK2*, *cysNC*	[29]

a The *cysN* and *cysC* genes of *Mtb* are fused into a single, bifunctional *cysNC* gene [22]. However, the transcript is often reported as *cysN* in microarray data.

SDS, sodium dodecyl sulfate.

Hypoxia is another environmental cue that has been correlated with the upregulation of genes from the sulfate assimilation pathway [23], [24]. Transcription of *cysD*, *cysNC*, *cysK2*, and *cysM* is induced by hypoxia, suggesting ATP sulfurylase and cysteine biosynthesis may facilitate bacterial adaptation to oxygen-limiting conditions. Because the granuloma has been shown to harbor regions of low oxygen tension [4], it is plausible that sulfur metabolism may be important in the transition to latency. The finding that reduced sulfur compounds are critical to the onset of chronic *Mtb* infection in mice lends additional support to this hypothesis [12].

Certain antibiotics have also been shown to affect the transcription of sulfur metabolism genes. Vancomycin, an inhibitor of peptidoglycan biosynthesis, induces expression of *cysK2*, *cysD*, and *cysNC* [29]. The *cysNC* gene is also induced by 5-chloropyrazinamide, a pyrazinamide analog that irreversibly inhibits fatty acid biosynthesis in vitro [20], [35]. Furthermore, *cysA1*, *cysT*, *cysW*, and *subI* are collectively induced by menadione, a synthetic vitamin K precursor that promotes the formation of reduced oxygen species [20], [36]. These data suggest that sulfur metabolism may modulate the bacterial response to certain drugs. Evaluating *Mtb* susceptibility to these drugs upon overexpression of related sulfur metabolism genes may reveal a more direct role for the encoded enzymes in antibiotic resistance.

Indeed, several studies point to a role for sulfur-containing compounds in bacterial detoxification pathways. In *Salmonella typhimurium*, cysteine biosynthesis is required for the enhanced antibiotic resistance of migrating swarm cells [37]. Similarly, dysregulation of cysteine metabolism in *Bacillus subtilis* and *Staphylococcus aureus* sensitizes the bacterial response to oxidative stress and tellurite exposure [38], [39]. Further, MSH exhibits well-documented protective effects against free radicals, potent oxidizing agents, alkylating agents, and certain antibiotics [14]–[16], [40], [41], though is essential for ethionamide susceptibility and was recently shown to be dispensable for *Mtb* growth [42]. Overall, these findings underscore the importance of bacterial sulfur metabolism to the detoxification of toxic species in the cell and are consistent with the enhanced expression of *Mtb* sulfur metabolism genes in response to oxidative stress and exposure to certain antibiotics.

### Transcriptional Regulators of Bacterial Sulfur Metabolism

**Figure 3:**
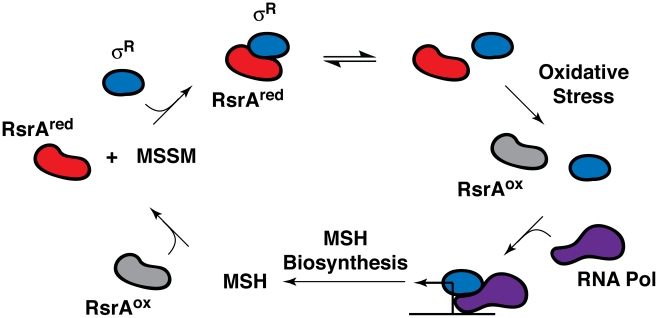
Feedback regulation of RsrA activity and SigR (σ^R^)-mediated transcription by MSH in *S. coelicolor*. The anti-sigma factor RsrA binds the alternative sigma factor σ^R^ in its reduced state (RsrA^red^), preventing the association of σ^R^ with RNA polymerase (RNA Pol). Under conditions of oxidative stress (e.g., hydrogen peroxide or diamide treatment), RsrA is oxidized (RsrA^ox^) and no longer binds σ^R^, enabling it to form a complex with RNA polymerase, which induces the transcription of MSH biosynthetic genes. The reduction of oxidized RsrA by MSH facilitates sequestration of σ^R^, which, in turn, suppresses MSH biosynthesis. Importantly, when oxidizing or alkylating agents deplete MSH levels, RsrA remains oxidized, which stimulates σ^R^-mediated transcription and MSH biosynthesis [45], [67].

In all likelihood, one or more transcriptional regulators mediate the transcriptional response of the sulfate assimilation pathway to extracellular signals. The alternative sigma factor SigH, which is induced by phagocytosis and stress conditions, including acidic pH and exposure to diamide, a thiol oxidizer, has been shown to regulate the transcription of several sulfur metabolism genes (e.g., *cysA1*, *cysT*, *cysW, cysD*, and *cysNC*) following diamide treatment [43]. SigH activity is regulated by the redox-sensitive anti-sigma factor RshA [44]. Interestingly, the alternative sigma factor SigR from *Streptomyces coelicolor*, a related actinomycete, is also induced by diamide and regulated by the redox-sensitive anti-sigma factor RsrA (Figure 3) [45]. SigR and RsrA have been shown to modulate MSH production in *S. coelicolor* in response to intracellular MSH levels [45]. The strong dependence of MSH biosynthesis on the sulfate assimilation pathway suggests that SigH and RshA might similarly regulate the biosynthesis of this ubiquitous thiol in mycobacteria.

In *Escherichia coli*, the majority of sulfate assimilation genes belong to the cysteine (*cys*) regulon that is positively regulated by the transcription factor CysB [46]. In the absence of inorganic sulfate, a second transcription factor, Cbl, induces a series of sulfate starvation-inducible (SSI) genes that coordinate the uptake and subsequent metabolism of organosulfur compounds for the generation of sulfite [47], [48]. Comparable transcriptional regulators have not yet been identified in mycobacteria.

Though the enzymes involved in sulfur metabolism are largely conserved between these two prokaryotes, there are some notable differences in the organization of their respective sulfate assimilation pathways that caution against using the transcriptional regulatory networks of *E. coli* as a blueprint for those of *Mtb*. For example, the reductive branch of the *Mtb* sulfate assimilation pathway emanates from APS, whereas *E. coli* initiates the biosynthesis of reduced sulfur metabolites from PAPS [17]. *Mtb* also encodes four eukaryotic-like sulfotransferases (*stf0*, *stf1*, *stf2*, and *stf3*), which are absent from *E. coli* [11], [49]. Further, *Mtb* encodes two independent pathways for cysteine biosynthesis: the canonical pathway, which utilizes L-serine (as in *E. coli*), and a novel route that utilizes *O*-phospho-L-serine [33], [50], [51]. While these distinctions do not rule out the possibility of a CysB-like master regulator in *Mtb*, they do suggest that the transcriptional regulation of mycobacterial sulfur-metabolizing enzymes may diverge considerably from that of *E. coli*. Additional studies are needed to elucidate the transcriptional machinery mediating the expression of these enzymes in *Mtb*. Notably, a functional analog of CysB was recently identified in *Corynebacterium glutamicum*, a gram-positive bacterium belonging to the same suborder as *Mtb*, which will likely facilitate the search for related proteins in mycobacteria [52].

## Biochemical Regulation of Sulfur Metabolism

### Sulfate Transporters

In addition to the various environmental conditions that modulate sulfur metabolism at the transcriptional level, several proteins regulate the metabolic pipeline by mediating the availability of sulfate and its flux through the sulfate assimilation pathway. Most notable are the sulfate transporters, which enable the uptake of extracellular sulfate. The primary sulfate permease of *Mtb* is an ABC transporter encoded by the *subI*-*cysTWA1* operon [53]. Genetic disruption of the *cysA1* gene in *M. bovis* completely inhibits sulfate uptake *in vitro* and renders the mutant auxotrophic for methionine, yet does not impair bacterial survival in mice [53]. This implies either a strong reliance on methionine transport in vivo, or the presence of other sulfate transporters that can compensate for the loss of SubI-CysTWA1 activity during infection. Notably, *Mtb* encodes two additional putative sulfate transporters, Rv1739c and Rv1707, whose genes are induced 24 h postinfection of activated macrophages [21], [54]. Rv1739c expression in *E. coli* has been shown to enhance sulfate uptake, though complementation of the *M. bovis cysA1* mutant with the *Rv1739c* gene is not sufficient to restore sulfate prototrophy [54]. In contrast, little is known about the *Rv1707* gene product. Assuming all three of these transporters are functional, it is possible they modulate sulfate uptake in response to discrete environmental signals.

### Sulfatases

**Figure 4:**
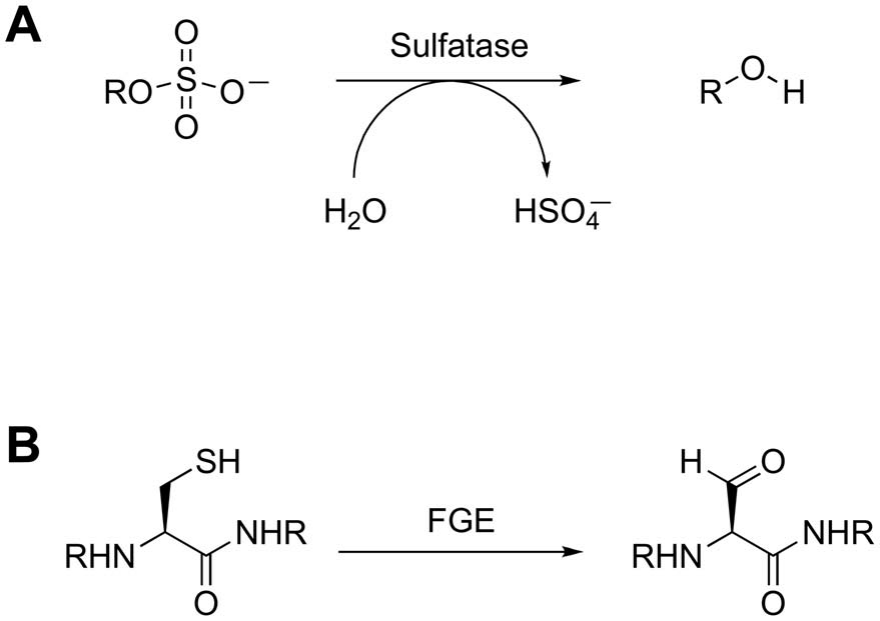
Sulfatase biochemistry. (A) Sulfatase-catalyzed hydrolysis of a sulfated metabolite. (B) Modification of the active site cysteine of type I sulfatases by FGE.

The sulfatases are a second class of proteins that likely contribute to the intracellular concentration of free sulfate. These enzymes catalyze the hydrolysis of sulfate esters from sulfated proteins, peptides, and small molecules (Figure 4A) [55]. Type I sulfatases are characterized by an active-site formylglycine residue that is critical for catalysis [56]. The formylglycine is either co- or post-translationally installed by a formylglycine-generating (FGE) enzyme via oxidation of a conserved cysteine (Figure 4B). The *Mtb* genome encodes six type I sulfatases, yet little is known about their biochemistry [56]. One of these, AtsG, was recently shown to possess arylsulfatase activity, but its native substrate has not been identified [57]. In the absence of FGE, *Mtb* exhibits residual sulfatase activity that may be attributed to FGE-independent type II and type III sulfatases [56]. It is possible that some of these enzymes are secreted and facilitate sulfate scavenging from the host. Alternatively, they may hydrolyze sulfate from endogenous metabolites to redirect the biosynthesis of sulfur-containing compounds in response to evolving cellular needs. In either scenario, these sulfatases are likely to regulate sulfur metabolism by influencing sulfate availability.

### The 3′-Phosphoadenosine-5′-Phosphatase CysQ

Another regulator of *Mtb* sulfur metabolism is CysQ, a 3′-phosphoadenosine-5′-phosphatase. Unlike sulfate permeases and sulfatases, whose activity directly influences the availability of free sulfate, CysQ has the ability to modulate the levels of intermediates that may affect flux through the sulfate assimilation pathway. Specifically, CysQ dephosphorylates 3′-phosphoadenosine 5′-phosphate (PAP), a byproduct of sulfotransferase reactions, and its sulfated counterpart, PAPS [58]. Since PAP inhibits at least one *Mtb* sulfotransferase [59], and PAPS accumulation is believed to be cytotoxic [60], the degradation of these pathway intermediates by CysQ is likely to promote sulfation and balance sulfur transactions in the cell. Interestingly, CysQ activity is inhibited by alkali metal cations in vitro, including physiological concentrations of sodium, and a homologous enzyme from *Streptococcus mutans* confers resistance to superoxide stress [58], [61]. These findings suggest that CysQ may be sensitive to changes in the ionic composition of the cytosol or to oxidative stress encountered during the course of infection. If so, CysQ may modulate sulfur metabolism in response to evolving environmental conditions.

## Molecular Mechanisms of Sulfur Metabolism Regulation

Small molecule regulation of sulfur metabolism is another important mechanism directing the biosynthesis of sulfur-containing metabolites in bacteria. Though this phenomenon has not been thoroughly investigated in *Mtb*, several paradigms have emerged from other bacteria that may prove functional in mycobacteria. While it is important to consider genus- and species-specific variations in sulfur metabolism when drawing such parallels [62], regulatory networks from other bacteria have often guided the discovery of comparable pathways in *Mtb* [63]–[65] and thus warrant further discussion.

In *S. typhimurium*, cysteine, sulfide, and thiosulfate have all been shown to inhibit sulfate assimilation [46]. Cysteine does so by inhibiting serine acetyltransferase, which catalyzes the formation of *O*-acetylserine, the biosynthetic precursor of cysteine. In contrast, sulfide and thiosulfate bind the CysB transcriptional regulator, repressing the transcription of genes that facilitate sulfide biosynthesis or in the case of thiosulfate, its uptake from outside the cell. In *E. coli*, APS has been shown to inhibit Cbl-mediated transcription, preventing the induction of SSI genes that facilitate sulfur metabolism in the absence of inorganic sulfate [66]. Thus, APS may serve as a molecular barometer of sulfate assimilation that modulates gene expression in response to sulfate availability. While similar feedback mechanisms remain to be elucidated in *Mtb*, they are likely to play an important role in regulating sulfur metabolism. Consistent with this hypothesis, transcription of the *cysDNC* operon of *Mtb* increases in response to sulfur limitation and decreases following treatment with exogenous cysteine [22].

MSH is also likely to trigger transcriptional regulation of sulfur-metabolizing enzymes in *Mtb*. As mentioned previously, the alternative sigma factor SigR from *S. coelicolor* has been shown to modulate the biosynthesis of MSH in response to the intracellular MSH concentration [45], [67]. The current model of MSH regulation revolves around the redox-sensitive anti-sigma factor RsrA, which binds SigR in its reduced state, sequestering the sigma factor and preventing it from binding RNA polymerase (Figure 3). MSH facilitates this activity by maintaining RsrA in its reduced state. Thus, when MSH is abundant, RsrA remains active, suppressing MSH biosynthesis. However, when thiol-reactive toxins overwhelm the cell's MSH supply, RsrA is no longer maintained in its reduced form, freeing SigR to induce MSH biosynthesis. Given the importance of MSH in maintaining the reducing environment of the cytosol and promoting resistance to toxic oxidants [68], it is likely that a similar regulatory network is in place to regulate its biosynthesis in mycobacteria.

## Identifying Nodes of Regulatory Convergence in *Mtb* Sulfur Metabolism

A global assessment of the transcriptional, biochemical, and molecular mechanisms of sulfur metabolism regulation described herein suggests nodes of regulatory convergence, or enzymes that are sensitive to multiple regulatory cues. These enzymes are apparently poised to tune flux through the sulfate assimilation pathway in response to substrate availability and a broad range of environmental signals. While many sulfur-metabolizing enzymes are promising drug targets (please see the review by Bhave et al. [6] for a comprehensive discussion), those that occupy key positions in the regulatory landscape may be particularly strong candidates for therapeutic intervention.

**Figure 5:**
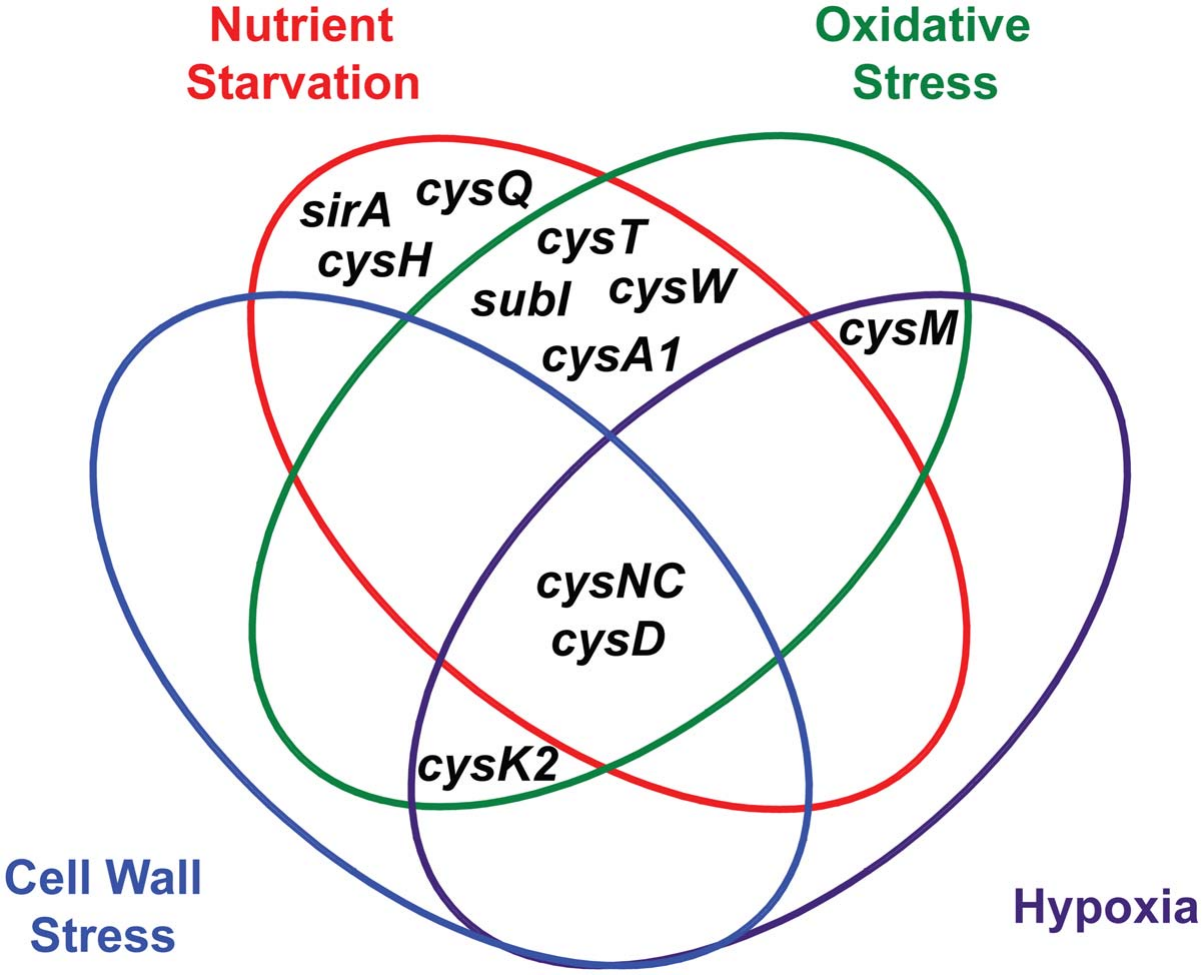
Venn diagram illustrating the convergent transcriptional regulation of *Mtb* sulfur metabolism genes by various conditions of environmental stress.

ATP sulfurylase and APS kinase, encoded by the *cysDNC* operon, are the most prominent regulatory nodes to emerge from this analysis. As illustrated in Figure 5, the *cysD* and *cysNC* genes are consistently upregulated by diverse environmental cues, including nutrient starvation [26], [27], hypoxia [23], [24], oxidative stress [21], [22], and cell wall stress [28], [29]. These genes are also induced upon phagocytosis [21], [25], a process that integrates many of these signals *in vivo*. Moreover, the catalytic activities of ATP sulfurylase and APS kinase make them particularly susceptible to biochemical regulatory cues. Together, these enzymes form the sulfate-activating complex (SAC) of *Mtb*, which converts free sulfate into PAPS [18]. Sulfate transporters, and possibly sulfatases, indirectly regulate this complex by determining sulfate availability in the cell. CysQ may also influence SAC activity by affecting the equilibrium between PAPS and APS. Finally, both cysteine and sulfate have been shown to modulate *cysDNC* transcription [22], indicating these genes are subject to small molecule regulation. In sum, ATP sulfurylase and APS kinase are highly regulated enzymes whose inhibition may critically impair *Mtb* sulfur metabolism.

Notably, ATP sulfurylase, a heterodimeric enzyme with both GTPase (CysN) and sulfurylase (CysD) domains, possesses several unique features that make it a particularly compelling drug target [6]. Since this enzyme catalyzes the first committed step in the *Mtb* sulfate assimilation pathway [18], its inhibition would eliminate flux through the pathway. Further, significant structural, mechanistic, and genetic features (e.g., the presence of a GTPase domain; the fusion of *cysN* with *cysC*) distinguish ATP sulfurylase from its human counterpart, which may facilitate the discovery of selective inhibitors [6], [18], [69]. Consistent with these findings, CysD was classified as a high-confidence drug target by an in silico target identification program following an extensive series of systems-, sequence-, and structure-based analyses [70].

Additional regulatory nodes suggested by the combined transcriptional data (Figure 5) are *cysK2*, *cysM*, and the *subI*-*cysTWA1* operon. Both *cysK2* and *cysM* are annotated as putative *O*-acetyl-L-serine sulfhydrylases (OAS), enzymes that catalyze the PLP-dependent biosynthesis of L-cysteine from *O*-acetyl-L-serine and a sulfur donor such as sulfide [34]. In fact, a third gene, *cysK1*, encodes the true OAS of *Mtb* [71]. CysM has been shown to catalyze an alternate cysteine biosynthetic pathway that uses *O*-phosphoserine as a substrate [33], [51], while the role of CysK2 in sulfur metabolism remains an enigma. Both *cysK2* and *cysM* are transcriptionally regulated by multiple environmental cues (Table 1) [21], [24], [29], [72]. Similarly, the *subI*-*cysTWA1* operon, which encodes the primary sulfate transport system of *Mtb*, is sensitive to diverse stress conditions [20], [21], [26], [27]. CysT, CysW, and CysK2 have also been classified as high-confidence drug targets [70]. Thus, these enzymes warrant further biochemical investigation and may prove attractive targets for the inhibition of *Mtb* sulfur metabolism.

## Conclusions and Perspectives

The sulfate assimilation pathway of *Mtb* is responsible for the biosynthesis of sulfur-containing metabolites that influence bacterial pathogenesis. The transcriptional regulation of this pathway in response to multifarious environmental cues, including those typically encountered in the phagosome, likely facilitates adaptation to host immune cells. In addition, several proteins that mediate the flux of sulfate through the pathway, such as sulfate permeases, sulfatases, and the phosphatase CysQ, modulate the biosynthesis of sulfur-containing compounds in response to the evolving metabolic demands of the cell. Finally, the potential for small molecule regulation of *Mtb* sulfur metabolism abounds; the metabolites produced by the sulfate assimilation pathway are themselves candidate regulators of this network, as evidenced by cysteine and MSH in other bacteria. Realizing the extent of this regulation remains an outstanding challenge in the field, as does identifying the transcriptional proteins that orchestrate this pathway's response to the bacterial environment. Characterization of CysK2 activity and the design of selective inhibitors targeting it and other nodes of regulatory convergence, particularly ATP sulfurylase, also warrant exploration. Addressing these aims will not only advance our understanding of sulfur metabolism in *Mtb*, but may also reveal a molecular linchpin whose inhibition could dismantle an essential metabolic pathway in this ubiquitous human pathogen.
